# Paper-Based Analytical Device for Zinc Ion Quantification in Water Samples with Power-Free Analyte Concentration

**DOI:** 10.3390/mi8040127

**Published:** 2017-04-18

**Authors:** Hiroko Kudo, Kentaro Yamada, Daiki Watanabe, Koji Suzuki, Daniel Citterio

**Affiliations:** Department of Applied Chemistry, Keio University, 3-14-1 Hiyoshi, Kohoku-ku, Yokohama 223-8522, Kanagawa, Japan; kudohiroko1224@keio.jp (H.K.); ymkn.z3@keio.jp (K.Y.); daik-diak@keio.jp (D.W.); suzuki@applc.keio.ac.jp (K.S.)

**Keywords:** colorimetry, analyte enrichment, PAD, inkjet printing, 3D-printing

## Abstract

Insufficient sensitivity is a general issue of colorimetric paper-based analytical devices (PADs) for trace analyte detection, such as metal ions, in environmental water. This paper demonstrates the colorimetric detection of zinc ions (Zn^2+^) on a paper-based analytical device with an integrated analyte concentration system. Concentration of Zn^2+^ ions from an enlarged sample volume (1 mL) has been achieved with the aid of a colorimetric Zn^2+^ indicator (Zincon) electrostatically immobilized onto a filter paper substrate in combination with highly water-absorbent materials. Analyte concentration as well as sample pretreatment, including pH adjustment and interferent masking, has been elaborated. The resulting device enables colorimetric quantification of Zn^2+^ in environmental water samples (tap water, river water) from a single sample application. The achieved detection limit of 0.53 μM is a significant improvement over that of a commercial colorimetric Zn^2+^ test paper (9.7 μM), demonstrating the efficiency of the developed analyte concentration system not requiring any equipment.

## 1. Introduction

Zinc ions (Zn^2+^) are a major metal contaminant in environmental water originating for example from mine drainage, industrial waste water, and galvanized steel pipes, among others. Environmental standards defining maximum allowable concentrations of Zn^2+^ are provided by various organizations. For instance, the WHO guideline for drinking water defines the standard value as 3 mg·L^−1^ (46 μM) [1]. In Japan, the maximum permissive concentration in drinking water of 1.0 mg·L^−1^ (15 μM) is set by the Ministry of Health, Labor, and Welfare [2]. In addition, the recommended water quality criteria for aquatic life provided by the EPA is even more strict (120 μg·L^−1^; 1.8 μM of Zn^2+^) [3]. Conventional analytical techniques such as inductively coupled plasma-mass spectrometry (ICP-MS) [4] and atomic absorption spectrometry (AAS) [5,6] enable selective detection of trace amounts of Zn^2+^. However, the necessity of sophisticated analytical instruments operated by trained specialists at relatively high running costs hampers their field use in routine environmental monitoring. 

In 2007, the Whitesides group has introduced microfluidic paper-based analytical devices (μPADs) as a novel chemical analysis platform [7]. Aside from advantages such as low-cost, light-weight, and safe disposability by incineration, the availability of capillary action allowing spontaneous sample transportation in paper channels has made μPADs simple, yet highly-functional analytical tools. From the original purpose of medical diagnosis [7], the application of μPADs has been expanded to a variety of fields [8,9,10,11,12,13,14], including environmental monitoring [15]. Examples of μPAD analysis of trace metal contaminants in environmental water include electrochemical lead detection [16], colorimetric iron detection [17], and a lateral flow immunoassay for cadmium detection [18]. Despite its importance, the demonstration of Zn^2+^ quantification on μPADs remains scarce. Although not being applied to environmental monitoring, electrochemical measurement of Zn^2+^ on a μPAD has been reported with a limit of detection of 1.0 μg·L^−1^ (15 nM) [19]. In an example of a colorimetric detection approach, Feng and co-workers have achieved the detection of 50 μM (3.3 mg·L^−1^) of Zn^2+^ with the aid of hierarchical cluster analysis (HCA) [20]. 

The current work demonstrates the colorimetric detection of Zn^2+^ on a paper-based analytical device equipped with a target analyte concentration system to achieve sufficient detection sensitivity for environmental monitoring. Although colorimetric methods offer an easily detectable signal observable by the naked eye, their limited detection sensitivity is a general issue for the quantification of low analyte concentrations [21]. Several reports describe target analyte concentration approaches on PADs, for example by using heating [22], ion concentration polarization [23], or a pumping system [24]. Most notably, Satarpai et al. have demonstrated the colorimetric detection of low levels of Pb^2+^ in environmental water by using a peristaltic pumping system [24]. In the current work, concentration of Zn^2+^ from a comparably large amount of sample has been achieved by simple stacking of multiple layers of cellulosic porous substrates and a filter paper layer for colorimetric detection. Compared to conventional μPADs, the overlaid highly liquid absorbent cellulose pads allow wicking of an enlarged sample volume (1 mL), enabling external power-free concentration of Zn^2+^ from the sample into the colorimetric detection area. In addition, the requirement for sample pretreatment has been overcome by integrating the necessary reagents into the sample application area. Most importantly, the influence of Cu^2+^, a major interfering metal ion of the used Zincon colorimetric indicator, has been eliminated up to a concentration of 50 μM. The developed PAD has been applied to Zn^2+^ quantification in tap water and river water samples. 

## 2. Materials and Methods

### 2.1. Reagents and Equipment

All reagents were used as received without further purification. ZnCl_2_, CaCl_2_, MgCl_2_, MnCl_2_, CuCl_2_, NiCl_2_, FeCl_3_, and standard solutions of Pb(NO_3_)_2_, Cd(NO_3_)_2_, and HgCl_2_ were purchased from Wako Pure Chemical Industries (Osaka, Japan). Tetramethylammonium hydroxide·5H_2_O (TMAOH), *N*-Tris(hydroxymethyl)methyl-3-aminopropanesulfonic acid (TAPS) and poly(diallyldimethylammonium chloride) (PDDA) were purchased from Sigma-Aldrich (St. Louis, MO, USA). Salicylaldoxime was obtained from TCI (Tokyo, Japan). 1-(2-Hydroxycarbonylphenyl)-5-(2-hydroxy-5-sulfophenyl)-3-phenylformazan, sodium salt (Zincon) was obtained from Dojindo (Kumamoto, Japan). Whatman No. 1 filter paper (460 mm × 570 mm) and CF7 absorbent pads (22 mm × 50 m) were purchased from GE Healthcare Life Sciences (Buckinghamshire, UK). Glass fiber (GFDX203000) and cellulose pads (GFCP103000) were purchased from Merck Milllipore (Darmstadt, Germany). Baking paper was purchased from Toyo Aluminium Ekco Products Co., Ltd. (Osaka, Japan). Ultrapure water ( >18 MΩ·cm) was obtained from a PURELAB flex water purification system (ELGA, Veolia Water, Marlow, UK) and used for the preparation of all solutions.

A Xerox ColorQube 8570 wax printer (Xerox, Norwalk, CT, USA) was used to print hydrophobic barriers designed in Adobe Illustrator CC software. An iP2700 inkjet printer (Canon, Tokyo, Japan) was used to deposit the colorimetric assay reagent and the polymeric additive (Zincon and PDDA). For this purpose, standard Canon ink cartridges have been cut open and the sponges inside were removed, followed by washing with copious amounts of ultrapure water. A Silhouette Cameo electronic knife blade cutting device (Silhouette, Lehi, UT, USA) in double cutting mode was used to cut glass fiber disks into the desired sizes. Hot lamination was performed on a QHE325 laminator (Meiko Shokai, Tokyo, Japan) set for plain copy paper and a total thickness of 100 μm. A 9000F MARK II color scanner (Canon, Tokyo, Japan) was used to acquire images for quantitative evaluation of colorimetric response and numerical color intensity values were measured by the Image J color analysis software (NIH, Bethesda, MD, USA). An Objet30 Prime (Stratasys, Eden Prairie, MN, USA) 3D-printer was used to fabricate the device holder designed on 123D design software (Autodesk, San Rafael, CA, USA). A potentiometric Ca^2+^ monitoring device (LAQUA twin) was purchased from Horiba (Kyoto, Japan). Zinc test paper was purchased from Kyoritsu Chemical-Check Lab., Corp. (Tokyo, Japan).

### 2.2. Investigation of Reaction Time of Cu^2+^-Salicylaldoxime Chelation

150 μL of aqueous CuCl_2_ solution (50 μM) and 150 μL of salicylaldoxime solution (7.15 mM) in TAPS/TMAOH buffer (pH 8.5, 400 mM) were mixed in a micro centrifuge tube. 200 μL of the mixed solution was added to a microtiter plate well containing 10 μL of Zincon solution (1.6 mM) immediately (for a reaction time of 0 min) or 1 min after mixing (for a reaction time of 1 min). Absorption spectra were acquired with a Varioskan^TM^ Flash multi spectra microplate reader (Thermo Fisher Scientific, Waltham, MA, USA). 

### 2.3. Device Fabrication

**Figure 1:**
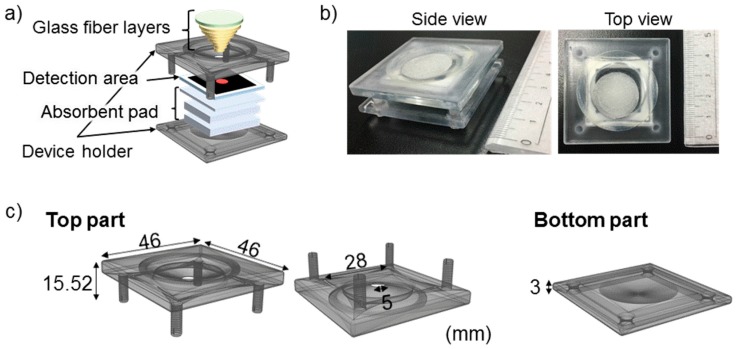
Design of the 3D-PAD for the detection of Zn^2+^ with integrated pretreatment and concentration systems: (**a**) schematic illustration; (**b**) actual photograph; (**c**) schematic illustration of disassembled device holder fabricated by 3D-printing.

As shown in Figure 1a,b, the device for the detection of low Zn^2+^ concentrations is composed of four sections: Glass fiber layers, colorimetric detection area, absorbent pad layers, and a device holder. The glass fiber layers were prepared by stacking seven glass fiber disks with different diameters (5, 6, 8, 11, 14, 17, and 20 mm from the bottom to top). Before stacking, salicylaldoxime solution (14.3 mM), and aqueous CuCl_2_ solution (100 μM) were pipetted onto the glass fiber disks (detailed conditions are summarized in Table 1), followed by drying at 40 °C for 1 h.

The detection area was prepared by using the wax printing technique [25,26]. Whatman No. 1 filter paper was cut into A4 size and fed into the ColorQube 8570 printer to print black wax on both sides of the filter paper in greyscale printing mode. A single detection area consists of an unmodified hydrophilic circular paper region (4 mm diameter) surrounded by a 20 × 20 mm^2^ solid black square-shaped hydrophobic wax region. Seventy detection areas were printed on a single A4 sheet. In order to achieve penetration of the printed wax throughout the paper thickness, the filter paper sandwiched by baking paper was passed through a hot laminator [27]. Finally, aqueous solutions of PDDA (1 wt %) and Zincon (1.6 mM) were inkjet-printed onto the hydrophilic circular area in 10 print cycles from the black and magenta ink reservoirs of the Canon printer, respectively.

Absorbent pad layers were prepared by using two types of cellulose pads (CFSP223000 and CF7). One sheet of CFSP223000 (cut into 30 × 30 mm^2^) and two sheets of CF7 (cut into 22 × 30 mm^2^) were manually piled up.

Finally, all porous substrate materials were assembled with the aid of a 3D-printed device holder composed of two parts (Figure 1c). The glass fiber pads were sequentially put onto the funnel-shaped pocket prepared on the top side of the holder. Patterned filter paper, CFSP223000, and CF7 were manually stacked and sandwiched by the screwed 3D-printed parts.

### 2.4. Detection and Quantification Method

Colorimetric detection was carried out by depositing 1 mL of sample onto the sample application area. After the entire liquid volume was absorbed by the absorbent pad layers (approximately 3 min after sample introduction), the detection filter paper was removed from the assembled device for color observation. For quantitative evaluation of colorimetric response, the detection filter paper was attached to a sheet of copy paper with double-sided tape and dried for 15 min at room temperature, followed by scanning at 600 dpi resolution and software-based digital color analysis of the photograph. 

## 3. Results and Discussion

### 3.1. Roles of the 3D-PAD Components

In order to achieve a low detection limit for Zn^2+^ with a simple colorimetric indicator on paper, a sample volume as large as 1 mL has been handled throughout this research, which is significantly larger than in common μPAD analyses (typically up to several tens of μL). Concentration of Zn^2+^ is achieved by the vertical passage of a large sample volume through the filter paper detection area with the Zincon colorimetric indicator. For ease of operation, the colorimetric detection of Zn^2+^ with an integrated concentration system requiring only a single sample application has been targeted in this work. The elaborated PAD consists of a filter paper-based colorimetric detection area and additional functional parts: glass fiber layers, absorbent pad layers, and device holder. 

#### 3.1.1. Filter Paper Detection Area

**Figure 2:**
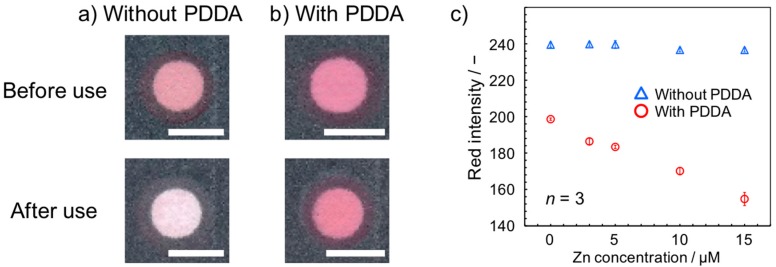
Immobilization of Zincon by poly(diallyldimethylammonium chloride) (PDDA). Scanned images of the detection areas before and after introduction of 1 mL water (**a**) in the absence and (**b**) presence of 10 cycles of inkjet deposited PDDA (scale bars: 4 mm); (**c**) quantitative evaluation of the colorimetric response to Zn^2+^ in the presence (red markers) and absence (blue markers) of PDDA (in this experiment, the glass fiber layers contain salicylaldoxime, buffer component, and CuCl_2_).

The colorimetric Zn^2+^ assay was carried out by using the Zincon indicator. For this purpose, Zincon was deposited by means of an inkjet printer onto a circular detection region (3.85 ± 0.03 mm diameter after wax perfusion; *n* = 5) prepared on filter paper. However, an additional immobilizing agent was necessary, because of the water-solubility of Zincon. With the current device design where up to 1 mL of sample liquid is passed through the colorimetric sensing layer, it is essential to immobilize the indicator and its Zn^2+^ complex formed during the assay. In our previous study, a cationically-charged nanoparticle has been employed for anchoring sulfonated colorimetric indicators onto a filter paper substrate [28]. The use of a piezoelectrically-actuated inkjet printer was inevitable for dispensing of the nanoparticle ink. In the present study, a water-soluble cationic polymer (PDDA) [29] was employed in place of the cationically-charged nanoparticle. Since aqueous solutions of PDDA are compatible with the thermally-actuated printer used, this allows for the colorimetric indicator and the immobilizing agent to be deposited by using a single inkjet printer. As compared to the state before sample liquid introduction, Zincon was completely washed away after introduction of 1 mL of water in the absence of PDDA (Figure 2a). With 10 printing cycles of PDDA solution (1 wt %), the pink color of Zincon was maintained after exposure to 1 mL of water (Figure 2b). It is postulated that the positive charge of the quaternary amino groups of PDDA allows electrostatic attractive interaction with both the negatively charged cellulosic paper surface [30] and the sulfonate groups of Zincon. The importance of the PDDA immobilizing agent has been quantitatively evaluated (Figure 2c). In contrast to the Zn^2+^ concentration-dependent color change with PDDA (red markers), the absence of PDDA resulted in no colorimetric response (blue markers). The increased red color intensity reflects the whiteness of the detection area due to washing out of the Zincon colorimetric indicator. 

The reproducibility of inkjet-deposition of the Zincon colorimetric indicator has been examined in a quantitative manner. For this purpose, digital color analysis of an inkjet printed PDDA-Zincon spot before exposure to a sample was performed. The measured red intensity was found to be 208 ± 1.47 (*n* = 34). The small standard deviation clearly indicates the reproducibility of reagent deposition by inkjet printing. 

#### 3.1.2. Glass Fiber Layers

The glass fiber layers on the top of the PAD serve as an inlet with integrated sample pretreatment function. Among various possible interferences in a Zincon-based Zn^2+^ assay, colorimetric response to copper ions (Cu^2+^) is most cumbersome owing to the overlapping working pH range (8.5−9.5 for Zn^2+^; 5.5−9.5 for Cu^2+^)[31] and similar maximum absorption wavelength of the colored metal-indicator complexes (620 nm for Zn^2+^, 600 nm for Cu^2+^)[31]. Salycilaldoxime, known as an effective chelating agent for Cu^2+^[32,33,34] as well as effective masking agent for other metal ions interfering the Zincon-based colorimetric Zn^2+^ detection including Fe^2+^[35,36,37] and Ni^2+^[35,37], has been deposited on the glass fiber layers together with the buffer components (TAPS/TMAOH, pH = 8.5). Additionally, CuCl_2_ has been placed on the topmost glass fiber layer for further suppressing interference from Cu^2+^ (details are discussed in Section 3.2). Glass fiber has been selected as the substrate material of choice for the sample pretreatment region, due to its ability of smooth reagent release.

#### 3.1.3. Absorbent Pad Layers

Cellulose pad materials (CFSP223000 and CF7) placed at the bottom of the PAD beneath the detection layer work as a “wicking pad” as seen in lateral flow immunoassay devices. Two sheets of CF7 cellulose pad provide high absorption capacity of waste sample. The CFSP223000 cellulose pad, placed between the detection filter paper layer and the CF7 pads, results in moderate sample transportation rate, ensuring sufficient reaction time for Zn^2+^ and Zincon. 

#### 3.1.4. Device Holder

There exist several approaches to fabricate μPADs composed of stacked multiple paper layers (3D-μPADs). In 2008, Martinez et al. first reported a 3D-μPAD assembled by using double-side adhesive tape and cellulose powder [38]. In 2011, the Crooks group utilized an aluminum housing to support an origami 3D-μPAD [39]. In 2012 and 2013, sprayed adhesive [40] and toner [41] have been employed to attach multiple layers of paper substrates. In the current study, a 3D-printed device holder has been prepared to assemble the 3D-PAD, since no modification of substrate surfaces is involved. The 3D-printed holder consists of two (top and bottom) parts fastened by screws (Figure 1c). The top part bears a funnel-shaped pocket to accommodate the seven layers of glass fiber substrates serving as an inlet and sample pretreatment region. 

### 3.2. Suppression of Cu^2+^ Interference

**Figure 3:**
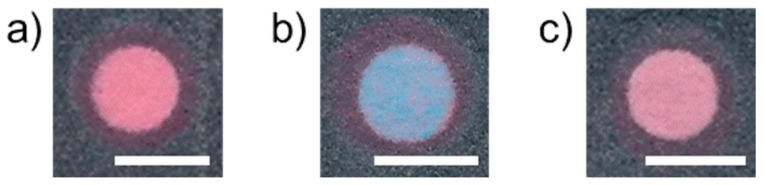
Effect of Cu^2+^ masking by salicylaldoxime: scanned images of the filter paper detection area after addition of 1 mL of (**a**) pure water on device with reagent impregnated glass fiber layers, (**b**) 50 μM aqueous CuCl_2_ solution on device without glass fiber layers, (**c**) 50 μM aqueous CuCl_2_ solution on a device with impregnated glass fiber layers (scale bars: 4 mm).

At first, the effect of Cu^2+^ masking by salicylaldoxime was confirmed by comparing the colorimetric response obtained for devices with and without the masking agent impregnated glass fiber layers. As compared to the pink color observed after application of a pure water sample (Figure 3a), the presence of 50 μM Cu^2+^ resulted in a color change of the detection area to blue (Figure 3b) in the absence of the glass fiber layers. On the other hand, the color change was significantly suppressed in the presence of the glass fiber layers impregnated with salicylaldoxime and the buffer components (Figure 3c), indicating successful Cu^2+^ masking. 

**Figure 4:**
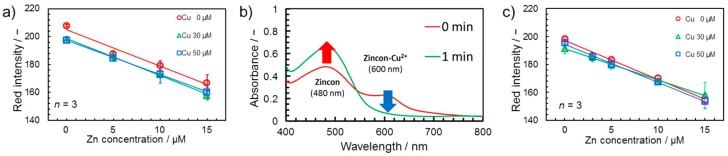
(**a**) Calibration curves for Zn^2+^ in the presence of 0, 30, and 50 μM of Cu^2+^ background obtained on devices with glass fiber layers impregnated with salicylaldoxime and buffer components. (**b**) Absorbance spectra in solution showing the delayed kinetics of Cu^2+^ masking by salicylaldoxime (detailed experimental conditions available in Section 2.2). (**c**) Calibration curves for Zn^2+^ in the presence of 0, 30, and 50 µM of Cu^2+^ background in the sample obtained on devices with glass fiber layers treated with salicylaldoxime, buffer components, and CuCl_2_.

However, deposition of salicylaldoxime and buffer components was not sufficient to allow accurate Zn^2+^ quantification in the presence of Cu^2+^. Typical calibration curves for Zn^2+^ still differed in the absence (0 μM) or presence (30, 50 μM) of a Cu^2+^ background in the sample (Figure 4a). Interestingly, no differences were observed between the presence of 30 and 50 μM of Cu^2+^. Based on the observed downward shift of the *y*-intercept of the calibration curve with identical slope upon the initial addition of Cu^2+^ only and no further shifts at increased Cu^2+^ background concentration, it was postulated that a constant amount of unmasked Cu^2+^ reaches the detection area despite the presence of the masking agent in the glass fiber areas. To evaluate the possible cause behind this experimentally observed behavior, a simple solution-based experiment was performed. Although the reported complex formation constant is higher for Cu^2+^-salicyladoxime (log *K* = 7.94) [42] compared to Cu^2+^-Zincon (log *K* = 7.5) [43,44], there is a difference in binding kinetics. As shown in Figure 4b, a significant absorbance peak at 600 nm, representing the Cu^2+^-Zincon complex, is observed immediately after mixing of reagents, while this peak is completely suppressed and the resulting spectra overlapping the metal free absorption spectrum of Zincon (480 nm) when allowing Cu^2+^ and salicylaldoxime to interact for 1 min before addition to the Zincon indicator solution. This indicates a fast interaction of Cu^2+^ ions with Zincon, followed by the slower displacement with the thermodynamically more stable salicyladoxime complex. The fact that the chelation of Cu^2+^ present in the sample solution by salicylaldoxime is not instantaneous results in a certain amount of free Cu^2+^ reaching the colorimetric detection area of the device, inducing a colorimetric signal. To reduce the shift in the calibration curve caused by this phenomenon, it was attempted to pre-deposit CuCl_2_ as a sample-independent source of Cu^2+^ on the topmost glass fiber layer. Calibration curves obtained using devices with pre-deposited Cu^2+^ (Figure 4c) demonstrate that, through this strategy, the influence of different Cu^2+^ background concentrations in samples (0, 30, and 50 μM) were finally significantly reduced.

### 3.3. Selectivity Study

#### 3.3.1. Primary Heavy Metal Contaminants

**Figure 5:**
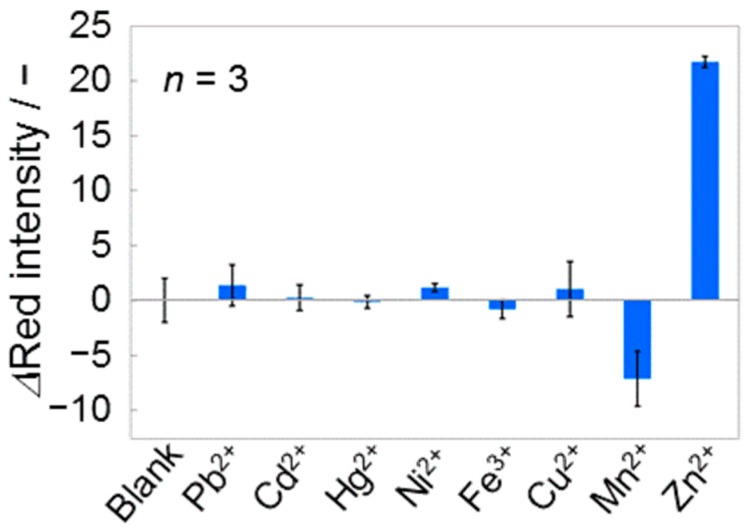
Selectivity of the Zn^2+^ detection device against other primary heavy metal contaminants. The graph shows colorimetric response to single heavy metals (Zn^2+^: 7.5 μM, other metals: 10 μM).

The selectivity of the Zn^2+^ detecting 3D-PAD has been evaluated using heavy metal ions known as primary contaminants in environmental water samples (Pb^2+^, Cd^2+^, Hg^2+^, Ni^2+^, Fe^3+^, Cu^2+^, and Mn^2+^). The metal ion samples have been prepared in water at 10 μM, whereas Zn^2+^ was tested at 7.5 μM (Figure 5). Among the tested heavy metal ions, the presence of Mn^2+^ exhibited significantly lowered red color intensity. This result is attributed to the inability of Mn^2+^ masking due to its poor binding property with salicylaldoxime [35], and the weaker absorbance of the Zincon-Mn^2+^ complexes compared to that of free Zincon [45]. 

#### 3.3.2. Influence of Ca^2+^

**Figure 6:**
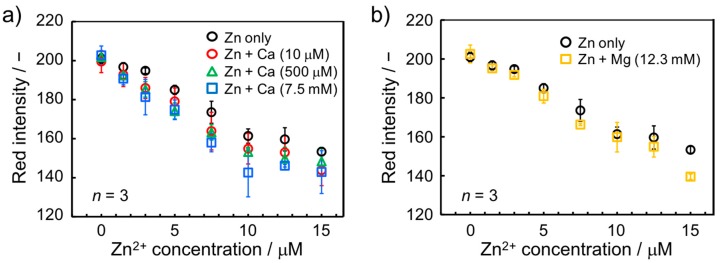
Zn^2+^ concentration-dependent response curves recorded in the presence of (**a**) Ca^2+^ and (**b**) Mg^2+^ ion background of various concentrations.

It is well known that Ca^2+^ ions undergo significant interaction with Zincon and that the chelation of Ca^2+^ induces UV/VIS spectral changes [31,45]. Nevertheless, this does not pose a problem for Zincon-based Zn^2+^ assays in solution, since selectivity can be readily achieved by the selection of a suitable measurement wavelength. Zn^2+^ and Ca^2+^ complexes with the indicator show distinguishable spectral behavior. In the case of a paper-based Zn^2+^ assay however, a specific wavelength selection is not achievable through visual inspection or simple colorimetric data analysis and therefore, the presence of Ca^2+^ interferes with Zn^2+^ detection. Considering the inability of selective Ca^2+^ masking and its abundance in environmental water samples, the effect of the presence of Ca^2+^ on quantitative Zn^2+^ detection with the current paper-based device was investigated. On the basis of the Japanese criteria for total water hardness of < 300 mg·L^−1^, [46] the investigated maximum Ca^2+^ concentration has been selected as 7.5 mM (300 mg·L^−1^). As expected, the presence of Ca^2+^ exhibited a significant influence on colorimetric Zn^2+^ detection (Figure 6a). On the other hand, Mg^2+^ showed no significant interference when present at concentration up to 12.3 mM (300 mg·L^−1^) (Figure 6b), reflecting the reported absence of UV/VIS spectral response of Zincon towards that cation [31,45]. The different calibration curves recorded in the presence of Ca^2+^ are attributed to its hypochromic spectral change [31], where Zincon-Ca^2+^ complexes exhibit decreased absorbance and thus show a weaker color intensity than metal-free Zincon. It was postulated that the decrease of the pink color due to the presence of Ca^2+^-bound Zincon leads to an increased sensitivity of the Zn^2+^ concentration-dependent calibration curve. On the other hand, the experimental results shown in Figure 6a demonstrate that Ca^2+^ background concentrations of 500 µM and 7.5 mM result in identical response curves for Zn^2+^. Therefore, Ca^2+^ concentration-independent colorimetric Zn^2+^ measurements with the paper-based device are achievable in water samples with a presence of 500 µM to 7.5 mM Ca^2+^ if calibration is performed accordingly. While this might not allow the application of the present device in all types of water samples, it can reasonably be assumed to be useful in common environmental water samples including tap water, as demonstrated in the following section. 

### 3.4. Application in Environmental Water Sample Matrix

**Figure 7:**
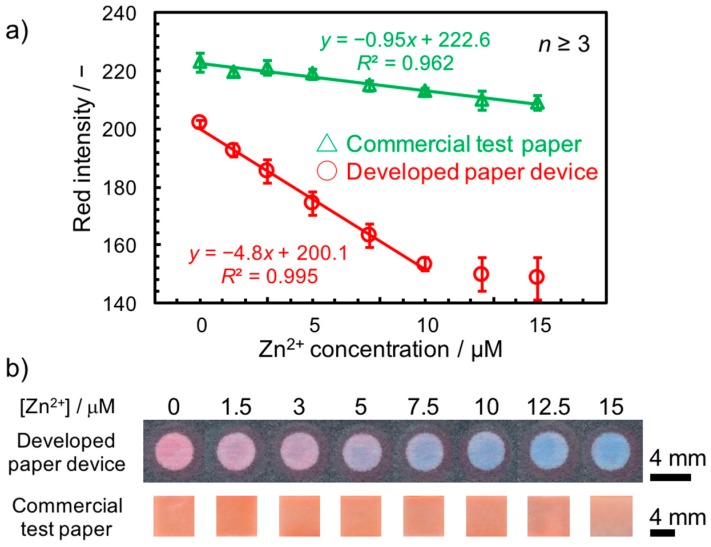
Comparison of Zn^2+^ detection with the paper-based device developed in this study and a commercial Zn^2+^ test strip. (**a**) Calibration curves for Zn^2+^ in a background of 500 μM Ca^2+^ targeting real sample analysis recorded with the paper-based device developed in this study (red line) and a commercial Zn^2+^ test strip (green line). (**b**) Scanned images of the Zn^2+^ testing area of the developed device (top row) and a commercial test strip (bottom row) at the corresponding Zn^2+^ concentrations in a background of 500 μM Ca^2+^.

Quantitative Zn^2+^ concentration measurements with the paper-based device developed in this study were performed in real sample matrices by spiking of 5.0 and 10.0 μM of Zn^2+^ into tap water and river water (Yagami River, Yokohama, Japan) samples. Recovery values were calculated based on a calibration curve obtained from aqueous Zn^2+^ solutions with a 500 μM Ca^2+^ background (red line in Figure 7) under the assumption that the Ca^2+^ concentration in the real sample matrix is within the range of 500 μM and 7.5 mM. This assumption was experimentally confirmed by direct measurements of Ca^2+^ in the collected samples using a potentiometric Ca^2+^ monitoring device. The recovery values summarized in Table 2 suggest that reasonable Zn^2+^ quantification was possible regardless of varying Ca^2+^ concentrations (0.825 mM for tap water, 1.65 mM for river water).

Finally, to demonstrate the superior sensitivity and lower detection limit of the current device compared to a common Zn^2+^ test paper, a calibration curve in the presence of Ca^2+^ was also recorded (green line in Figure 7) with a commercially available test strip. In comparison with the conventional Zn^2+^ test paper based on the same Zincon indicator, the current device exhibited a significantly improved sensitivity (slope of the calibration curves: −4.8 for the current device; −0.95 for the test paper). The achieved detection limit of 0.53 μM allows the detection of Zn^2+^ even below the environmental standard provided by the EPA (1.8 μM), in contrast to the much higher detection limit obtained with a Zn^2+^ test paper currently on the market (9.7 μM). The material cost has been calculated as $0.398 for a single 3D-PAD and $14.0 for the 3D-printed device holder (calculation details on 3D-PAD and 3D-printed device holder are available in Tables S1 and S2 of the Supplementary Materials). Considering their relatively high-cost, re-use of the 3D-printed device holders is desired in practical use. 

## 4. Conclusions

This work has demonstrated concentration and colorimetric detection of Zn^2+^ on a paper-based analytical device. Integration of multiple layers of porous substrates, including glass fiber for sample pre-treatment and cellulose pads for liquid absorption, enables power-free concentration of the analyte from a single sample application. Importantly, the influence of Cu^2+^ possessing an identical working pH range and colorimetric response with Zn^2+^ has been significantly suppressed with the aid of the salicylaldoxime masking agent. Although successful application to Zn^2+^ determination in environmental water matrix has been achieved, challenges remain in real sample analysis under more extreme conditions, such as high heavy metal ion contamination or extremely low concentration of Ca^2+^. However, it is believed that the current analyte concentration approach requiring no electrical power source is compatible with the inherent simplicity of paper-based analytical devices. We hope that the current system helps to overcome the inherently limited sensitivity of simple colorimetric devices and contributes to the expanded applicability of paper-based analytical devices for rapid and simple colorimetric detection of trace metals. 

## Figures and Tables

**Table 1:** Summary of reagents added onto the glass fiber layers.

Glass Fiber Disk Diameter	Added Reagent	Pipetting Amount
5 mm	14.3 mM of salicylaldoxime in buffer solution (TAPS/TMAOH, pH 8.5, 400 mM)	10.0 μL
6 mm		14.4 μL
8 mm		25.6 μL
11 mm		50.0 μL
14 mm		80.0 μL
17 mm		116.0 μL
20 mm	100 μM of aqueous CuCl_2_ solution	160.0 μL

**Table 2:** Quantification of Zn^2+^ in spiked tap water and river water samples (*n* = 3).

Sample	Added Zn/μM	Found Zn/μM	Recovery/%
Tap water	0	Not detectable	-
	5	5.76 ± 1.04	115
	10	8.93 ± 1.08	89
River water	0	Not detectable	-
	5	6.48 ± 0.72	129
	10	10.6 ± 0.47	106

## Supplementary Materials

The following are available online at www.mdpi.com/2072-666X/8/4/127/s1; Table S1: Material cost estimation for the developed 3D-PAD. Table S2: Material cost estimation for the 3D-printed device holder.

## References

[1] World Health Organization (2011). Guidelines for Drinking-Water Quality.

[2] The Ministry of Health, Labour and Welfare of Japan, Drinking Water Quality Standards. http://www.mhlw.go.jp/english/policy/health/water_supply/dl/4a.pdf.

[3] EPA, National Recommended Water Quality Criteria—Aquatic Life Criteria Table. https://www.epa.gov/wqc/national-recommended-water-quality-criteria-aquatic-life-criteria-table.

[4] Goullé J.-P., Mahieu L., Castermant J., Neveu N., Bonneau L., Lainé G., Bouige D., Lacroix C. (2005). Metal and metalloid Multi-Elementary ICP-MS validation in whole blood, plasma, urine and hair: Reference values. Forensic Sci. Int..

[5] Jorgensen Cassella R., Teixeira Bitencourt D., Garcia Branco A., Luis Costa Ferreira S., Santiago de Jesus D., Souza de Carvalho M., Erthal Santelli R. (1999). On-line preconcentration system for flame atomic absorption spectrometry using unloaded polyurethane foam: Determination of zinc in waters and biological materials. J. Anal. At. Spectrom..

[6] Taylor A., Branch S., Halls D.J., Owen L.M.W., White M. (2000). Atomic Spectrometry Update: Clinical and biological materials, foods and beverages. J. Anal. At. Spectrom..

[7] Martinez A.W., Phillips S.T., Butte M.J., Whitesides G.M. (2007). Patterned paper as a platform for inexpensive, low-volume, portable bioassays. Angew. Chem. Int. Ed..

[8] Martinez A.W., Phillips S.T., Whitesides G.M., Carrilho E. (2010). Diagnostics for the developing world: Microfluidic paper-based analytical devices. Anal. Chem..

[9] Yetisen A.K., Akram M.S., Lowe C.R. (2013). Paper-based microfluidic point-of-care diagnostic devices. Lab Chip.

[10] Hu J., Wang S., Wang L., Li F., Pingguan-Murphy B., Lu T.J., Xu F. (2014). Advances in paper-based point-of-care diagnostics. Biosens. Bioelectron..

[11] Cate D.M., Adkins J.A., Mettakoonpitak J., Henry C.S. (2015). Recent developments in paper-based microfluidic devices. Anal. Chem..

[12] Yamada K., Henares T.G., Suzuki K., Citterio D. (2015). Paper-based inkjet-printed microfluidic analytical devices. Angew. Chem. Int. Ed..

[13] Lopez-Marzo A.M., Merkoci A. (2016). Paper-based sensors and assays: A success of the engineering design and the convergence of knowledge areas. Lab Chip.

[14] Xu Y., Liu M., Kong N., Liu J. (2016). Lab-on-paper Micro- and nano-analytical devices: Fabrication, modification, detection and emerging applications. Microchim. Acta.

[15] Meredith N.A., Quinn C., Cate D.M., Reilly T.H., Volckens J., Henry C.S. (2016). Paper-based analytical devices for environmental analysis. Analyst.

[16] Nie Z., Nijhuis C.A., Gong J., Chen X., Kumachev A., Martinez A.W., Narovlyansky M., Whitesides G.M. (2010). Electrochemical sensing in paper-based microfluidic devices. Lab Chip.

[17] Asano H., Shiraishi Y. (2015). Development of paper-based microfluidic analytical device for iron assay using photomask printed with 3D printer for fabrication of hydrophilic and hydrophobic zones on paper by photolithography. Anal. Chim. Acta.

[18] López Marzo A.M., Pons J., Blake D.A., Merkoçi A. (2013). All-integrated and highly sensitive paper based device with sample treatment platform for Cd^2+^ immunodetection in drinking/tap waters. Anal. Chem..

[19] Ruecha N., Rodthongkum N., Cate D.M., Volckens J., Chailapakul O., Henry C.S. (2015). Sensitive electrochemical sensor using a graphene-polyaniline nanocomposite for simultaneous detection of Zn (II), Cd (II), and Pb (II). Anal. Chim. Acta.

[20] Feng L., Li X., Li H., Yang W., Chen L., Guan Y. (2013). Enhancement of sensitivity of paper-based sensor array for the identification of heavy-metal ions. Anal. Chim. Acta.

[21] Yamada K., Shibata H., Suzuki K., Citterio D. (2017). Toward practical application of paper-based microfluidics for medical diagnostics: State-of-the-art and challenges. Lab Chip.

[22] Wong S.Y., Cabodi M., Rolland J., Klapperich C.M. (2014). Evaporative concentration on a paper-based device to concentrate analytes in a biological fluid. Anal. Chem..

[23] Yeh S.-H., Chou K.-H., Yang R.-J. (2016). Sample pre-concentration with high enrichment factors at a fixed location in paper-based microfluidic devices. Lab Chip.

[24] Satarpai T., Shiowatana J., Siripinyanond A. (2016). Paper-based analytical device for sampling, on-site preconcentration and detection of ppb lead in water. Talanta.

[25] Carrilho E., Martinez A.W., Whitesides G.M. (2009). Understanding wax printing: A simple micropatterning process for paper-based microfluidics. Anal. Chem..

[26] Lu Y., Shi W., Jiang L., Qin J., Lin B. (2009). Rapid prototyping of paper-based microfluidics with wax for low-cost, portable bioassay. Electrophoresis.

[27] Tenda K., Ota R., Yamada K., Henares T., Suzuki K., Citterio D. (2016). High-resolution microfluidic paper-based analytical devices for sub-microliter sample analysis. Micromachines.

[28] Henares T.G., Yamada K., Takaki S., Suzuki K., Citterio D. (2017). “Drop-Slip” bulk sample flow on fully inkjet-printed microfluidic paper-based analytical device. Sens. Actuators B Chem..

[29] Rattanarat P., Dungchai W., Cate D.M., Siangproh W., Volckens J., Chailapakul O., Henry C.S. (2013). A Microfluidic paper-based analytical device for rapid quantification of particulate chromium. Anal. Chim. Acta.

[30] Yamada K., Henares T.G., Suzuki K., Citterio D. (2015). Distance-based tear lactoferrin assay on microfluidic paper device using interfacial interactions on surface-modified cellulose. ACS Appl. Mater. Interfaces.

[31] Säbel C.E., Neureuther J.M., Siemann S. (2010). A Spectrophotometric method for the determination of zinc, copper, and cobalt ions in metalloproteins using Zincon. Anal. Biochem..

[32] Lucia M., Campos A.M., van den Berg C.M.G. (1994). Determination of copper complexation in sea water by cathodic stripping voltammetry and ligand competition with salicylaldoxime. Anal. Chim. Acta.

[33] Shibukawa M., Shirota D., Saito S., Nagasawa S., Saitoh K., Minamisawa H. (2010). Simple spectrophotometric determination of trace amounts of zinc in environmental water samples using aqueous biphasic extraction. Bunseki Kagaku.

[34] Mehio N., Ivanov A.S., Williams N.J., Mayes R.T., Bryantsev V.S., Hancock R.D., Dai S. (2016). Quantifying the binding strength of salicylaldoxime-uranyl complexes relative to competing salicylaldoxime-transition metal ion complexes in aqueous solution: A combined experimental and computational study. Dalton Trans..

[35] Burger K., Egyed I. (1965). Some theoretical and practical problems in the use of organic reagents in chemical analysis—V: Effect of electrophilic and nucleophilic substituents on the stability of salicylaldoxime complexes of transition metals. J. Inorg. Nucl. Chem..

[36] Thorpe J.M., Beddoes R.L., Collison D., Garner C.D., Helliwell M., Holmes J.M., Tasker P.A. (1999). Surface coordination chemistry: Corrosion inhibition by tetranuclear cluster formation of iron with salicylaldoxime. Angew. Chem. Int. Ed..

[37] Smith A.G., Tasker P.A., White D.J. (2003). The structures of phenolic oximes and their complexes. Coord. Chem. Rev..

[38] Martinez A.W., Phillips S.T., Whitesides G.M. (2008). Three-dimensional microfluidic devices fabricated in layered paper and tape. Proc. Natl. Acad. Sci. USA.

[39] Liu H., Crooks R.M. (2011). Three-dimensional paper microfluidic devices assembled using the principles of origami. J. Am. Chem. Soc..

[40] Lewis G.G., DiTucci M.J., Baker M.S., Phillips S.T. (2012). High throughput method for prototyping Three-dimensional, paper-based microfluidic devices. Lab Chip.

[41] Schilling K.M., Jauregui D., Martinez A.W. (2013). Paper and toner three-dimensional fluidic devices: Programming fluid flow to improve point-of-care diagnostics. Lab Chip.

[42] Das A.K. (1990). Astatistical aspects of the stabilities of ternary complexes of cobalt(II), nickel(II), copper(II) and zinc(II) involving aminopolycarboxylic acids as primary ligands and salicylaldoxime as a secondary ligand. Trans. Metal Chem..

[43] Cano-Raya C., Fernández-Ramos M.D., Capitán-Vallvey L.F. (2006). Fluorescence resonance energy transfer disposable sensor for copper (II). Anal. Chim. Acta.

[44] Lou X., Zhang L., Qin J., Li Z. (2008). An alternative approach to develop a highly sensitive and selective chemosensor for the colorimetric sensing of cyanide in water. Chem. Commun..

[45] Hilario E., Romero I., Celis H. (1990). Determination of the physicochemical constants and spectrophotometric characteristics of the metallochromic Zincon and its potential use in biological systems. J. Biochem. Biophys. Methods.

[46] The Ministry of Health, Labour and Welfare of Japan Revision of Drinking Water Quality in Japan. http://www.mhlw.go.jp/topics/bukyoku/kenkou/suido/kijun/dl/k34.pdf.

