# Supplementary material for: Paper-Based Analytical Device for Zinc Ion Quantification in Water Samples with Power-Free Analyte Concentration
Source: Micromachines (Basel). 2017 Apr 18;8(4):127. doi: 10.3390/mi8040127 (PMC6189703; doi:10.3390/mi8040127)
Supplement: Supplementary file 1 [file micromachines-08-00127-s001.pdf]

Paper-Based Analytical Device for Zinc Ion Quantification in Water Samples with Power-Free Analyte Concentration

Hiroko Kudo, Kentaro Yamada, Daiki Watanabe, Koji Suzuki, and Daniel Citterio

Table S1. Material cost estimation for single 3D-PAD.

Material	Cost Per Device
GFDX203000 glass fiber	$0.133
CF7 absorbent pad	$0.175
GFCP103000 cellulose pad	$0.0583
Whatman No. 1 filter paper	$0.00983
Zincon	$0.00000644
Salicylaldoxime	$0.000657
TAPS	$0.0116
TMAOH	$0.01016
Copper (II) chloride	$0.000000125
Total	$0.398

Table S2. Material cost estimation for the 3D-printed device holder.

Material	Cost Per Holder
FullCure810 VeroClear	$9.70
FullCure705 Resin Support	$4.33
Total	$14.0

© 2017 by the authors; licensee MDPI, Basel, Switzerland. This article is an open access article distributed under the terms and conditions of the Creative Commons Attribution (CC BY) license (http://creativecommons.org/licenses/by/4.0/).
